# Investigating the Microstructural and Mechanical Properties of Novel Ternary Reinforced AA7075 Hybrid Metal Matrix Composite

**DOI:** 10.3390/ma15155303

**Published:** 2022-08-01

**Authors:** Afnan Haider Khan, Syed Ahmad Ali Shah, Farheen Umar, Uneeb Noor, Rizwan Mahmood Gul, Khaled Giasin, Muhammad Aamir

**Affiliations:** 1 Department of Mechanical Engineering, University of Engineering and Technology, Mardan 23200, Pakistan; 2 Department of Mechanical Engineering, CECOS University of IT and Emerging Sciences, Peshawar 25000, Pakistan; ahmad.shah.me-2018a@cecosian.edu.pk (S.A.A.S.); uneeb.noor.me-2018a@cecosian.edu.pk (U.N.); 3 Department of Physics, University of Peshawar, Peshawar 25000, Pakistan; farheenumarf@uop.edu.pk; 4 Department of Mechanical Engineering, University of Engineering and Technology, Peshawar 25000, Pakistan; rgul@uetpeshawar.edu.pk; 5 School of Mechanical and Design Engineering, University of Portsmouth, Portsmouth PO1 3DJ, UK; khaled.giasin@port.ac.uk; 6 School of Engineering, Edith Cowan University, Joondalup 6027, Australia; m.aamir@ecu.edu.au

**Keywords:** AA7075, hybrid metal matrix composites, silicon carbide, rice husk ash, carbonized eggshell

## Abstract

This study investigates the comparison of the microstructural and mechanical properties of a novel ternary reinforced AA7075 hybrid metal matrix composite. Four samples, including AA7075 (base alloy), AA7075-5wt %SiC (MMC), AA7075-5wt %SiC-3wt %RHA (s-HMMC), and AA7075-5wt %SiC-3wt %RHA-1wt %CES (n-HMMC) were developed using the stir casting liquid metallurgy route, followed by the heat treatment. The experimental densities corresponded with the theoretical values, confirming the successful fabrication of the samples. A minimum density of 2714 kg/m^3^ was recorded for the n-HMMC. In addition, the highest porosity of 3.11% was found for n-HMMC. Furthermore, an increase of 24.4% in ultimate tensile strength and 32.8% in hardness of the n-HMMC was recorded compared to the base alloy. However, its ductility and impact strength was compromised with the lower values of 5.98% and 1.5 J, respectively. This was confirmed by microstructural analysis, which reveals that n-HMMC has mixing issues and forms agglomerates in the matrix, which served as the potential sites of stress concentration leading to low impact strength and ductility. Nevertheless, the hybrid composites showed superior mechanical properties over the MMC and its base alloy.

## 1. Introduction

Metal Matrix Composites (MMCs) have widespread applications in aerospace, defense, marine, and automotive industries due to their high strength-to-weight ratio, good wear resistance, lower corrosion, and good stability at higher temperatures [1,2,3,4,5,6]. The literature has confirmed that the mechanical, tribological, and thermal properties of alloys are further improved by the addition of the reinforcements, such as Titanium Carbide (TiC), Silicon Carbide (SiC), Boron Carbide (B_4_C), Alumina (Al_2_O_3_), Graphite, Silica (SiO_2_), Silicon Nitride (Si_3_N_4_), and Carbon nanotubes (CNTs) [1,7,8,9,10,11]. The three main routes of developing MMCs include solid-state (powder metallurgy), liquid-state (liquid Metallurgy), and deposition processes [12]. In the case of the liquid-state process, reinforcement is added to the alloy in its molten state, usually in the presence of inert gas. The powder metallurgy route involves the consolidation and fusion of reinforcing particulates (powder) through sintering into a solid metal matrix under higher pressures and temperatures below the melting point in the inert environment. Finally, the deposition technique involves the deposition of the reinforcements onto the matrix either by the physical vapor deposition or spray deposition method. Other methods include squeeze casting, pressure infiltration, pressure-less infiltration, friction stir processing (FSP), and ultrasonic-assisted casting [13,14]. 

Alongside the efforts to reduce the cost of synthesis and the processing of MMCs and Hybrid Metal Matrix Composites (HMMCs), modern engineers and material scientists have shown an interest in improving their mechanical and tribological properties by adding cheaper and green reinforcements. Therefore, reinforcing materials can be broadly classified into synthetic ceramic particulates, industrial wastes, and derivatives of agricultural waste. Among the derivatives of the agricultural waste, rice husk ash (RHA), corn stalk ash (CSA), groundnut shell, coconut shell, cow horn, bagasse, bamboo leaf ash (BLA), and corn cob ash (CCA) are the regularly used and cost-effective reinforcements for the development of MMCs [15]. However, studies have shown the significance of HMMCs over single reinforced MMC in recent times. This is because HMMCs have two or more different reinforcements in either the same or different physical forms, such as whiskers, particles, and fibers [16,17,18,19]. 

Due to its high strength-to-weight ratio, the 7xxx series of aluminum alloys are the preferred choice in aircraft, space, and military industries [20]. One of the most commonly used members of this series is the aluminum alloy AA7075 [21]. However, despite the high strength-to-weight ratio and better fatigue properties, the applications of AA7075 are limited because of its low wear resistance, fair tribological properties, average machinability, and higher cost [22]. The development of aluminum alloy metal matrix composites (AA-MMCs) using synthetic ceramic reinforcements, such as TiC, SiC, and Al_2_O_3_, has shown promising results in improving the tensile strength and hardness of AA7075 [23,24]. Wu et al. [25] reported that a decrease in the particle size of the B_4_C in Al7075/B_4_C composites resulted in higher yield strength and fracture strength. Recently, research on hybrid metal matrix composites has gained popularity, aiming to achieve superior mechanical and tribology properties at a reduced cost, using natural and/or synthetic waste materials. Baradeswaran et al. [26] investigated the mechanical and wear behavior of AA6061 and AA7075 hybrid composites reinforced with 10wt % B_4_C and 5wt % graphite, developed via a liquid route. The hardness, % elongation (%EL), and the wear resistance of the hybrid alloys were improved compared to the base alloys. Kumar et al. [27] developed an A356/(fly-ash + red mud) hybrid metal matrix surface composite (HMMSC) via the stir friction process (SFP). They reported the superior mechanical and tribological properties of HMMSC A356 over the as-casted and SFP A356 alloy. However, the ductility has been reportedly compromised in the HMMSC with respect to the FSP A356. Chechi et al. [28] investigated the microstructural and mechanical properties of the novel combination of AA6061/SiC/FA/Gr HMMC prepared via a liquid metallurgy process. Higher tensile strength and hardness were reported for the HMMC. However, embrittlement was increased due to which the reduction in %EL was reported. Arora and Sharma [29] performed a comparative study of AA6351 reinforced with SiC and RHA up to 8wt % each. The hardness and tensile strength of AA6351/SiC were reported to increase by 21% and 18%, respectively, compared to the AA6351/RHA composite. Singh et al. [30] fabricated ZA-27 hybrid metal matrix composites reinforced with lamb bone ash (LBA) and boron carbide (B_4_C) using the stir casting approach. They reported an increase in the tensile strength, compressive strength, and hardness of the HMMCs to the maximum value of 61.08%, 24.40%, and 41.12%, respectively, in comparison to its base alloy. However, the ductility and impact strength of hybrid composites were reduced. Tejyan et al. [31] used a stir casting approach to develop Al-6063-based HMMCs using SiC and Neem leaf ash (NLA) as reinforcing agents. The highest hardness, tensile, and impact strength was reported for 6wt % SiC with 4wt % NLA. Manikandan et al. [32] compared the mechanical, microstructural, and tribological properties of B_4_C and Cow dung ash (CDA) reinforced AA-7075 HMMC with the base alloy, prepared via two-stage stir casting. They reported improvement in the mechanical and tribological properties of the HMMC except for the impact strength, which was slightly compromised. The maximum hardness and flexure strength in the HMMC were reported to increase by 38% and 12%, respectively, in comparison to the base alloy.

Recently, researchers have shown progress toward the use of HMMCs in real-world engineering applications. Gupta et al. [33] prepared HMMC of Al-7.1Si (LM27) reinforced with sillimanite and rutile in the weight ratio of 1:1 via stir casting liquid metallurgy route for the brake rotor application. Rockwell hardness of the HMMC was reported to be 91 ± 3, which is comparable with commercially available cast iron. Similarly, at 15wt % of reinforcement, a drop of nearly 52% in the wear rate was reported in the HMMC in comparison to its base alloy, which was comparable to the results of the commercially available brake rotor material. Tan et al. [34] prepared lightweight hybrid metal matrix composite A357/SiC/AA6082 brake discs through FSP for the city rail vehicle under the city environmental conditions. It was reported that the disc brake successfully passed the dynamometer breaking test for more than 1000 breaking test cycles. Sharma et al. [35] summarized that HMMCs have better overall properties than their base alloy and MMCs. However, embrittlement has been reported to increase in many cases with the addition of reinforcements. Dhanesh et al. [36], in their review article, concluded that the aluminum-based hybrid metal matrix composites show superior mechanical, wear, and microstructural properties up to a certain saturation level of the reinforcements as compared to the base alloy and MMCs.

Rice husk and eggshells are natural waste products that can be utilized as reinforcing agents in composite materials [37,38,39]. According to the United Nations (UN) Food and Agriculture Organization (FAO), 769.9 million tons of rice were produced globally in 2018. Rice husk accounts for about 20wt % of the total rice production, from which it can be calculated that 153.98 million tons of rice husk waste were produced in 2018. Similarly, around 150 kilotons of eggshell waste are produced in the United States alone, whereas the global eggshell waste is around 7.2 million tons [40]. Different researchers have used these waste materials as reinforcements to develop MMCs and HMMCs. Verma and Vettivel [41] reported an increase in the hardness (HV 121) and compression strength (563 MPa) of AA7075 HMMC at 5wt % B_4_C/5wt % RHA as compared to its base alloy and MMCs. However, the tensile strength of HMMCs was lower than the MMC but higher than the base alloy. Alaneme and Sanusi [42] investigated the mechanical properties, microstructure, and wear behavior of the alumina, RHA, and graphite (Gr)-reinforced Al-Mg-Si hybrid alloy produced through two-step stir casting. They reported a decrease in the hardness with the increasing amount of RHA and graphite. Higher tensile strength of the composites with 0.5wt % Gr and up to 50% RHA was reported than those without graphite. The addition of RHA as reinforcement is not only limited to the alloys. Al-Alwan et al. [43] recently used RHA as a partial replacement of the ordinary Portland cement in the concrete. They reported a 9%, 11%, and 4% increase in the flexure, compression, and tensile strength of the concrete, respectively. Similarly, other researchers have also reported RHA to be a promising reinforcing agent to improve the overall performance of ceramics [44,45,46]. In addition to RHA, eggshell ash also has a lower density with good potential to be used as reinforcement in developing the metal matrix composite materials with improved overall properties and lower weight. Arunkumar and Senthil Kumar [47] investigated the tribological properties of two HMMCs; Al7075 + Al_2_O_3_ + SiC and Al7075 + Al_2_O_3_ + Eggshell, prepared via the stir casting technique. They reported highest value of hardness 197 Hv with 6% eggshell powder. Similarly, the wear properties of the base alloy were also improved due to the self-lubricating effect induced by the eggshell powder. Singh et al. [48] investigated the effect of eggshell ash (ESA) and boron carbide (0–5wt %) on the microstructural and mechanical properties of ZA-27 HMMCs. Improved hardness, tensile, and compressive strength were reported with the addition of the reinforcements. However, impact strength was compromised due to embrittlement and plastic deformation. Daud and Mohamad [49] reported an increase in the hardness and porosity upon the addition of eggshells as reinforcement to the aluminum matrix. Recently Gupta et al. [50] investigated RHA and carbonized eggshell (CES)-reinforced AA7075 composites for dry sliding friction and wear behavior. The sample with the highest CES content of 5wt % was reported to have maximum porosity, maximum wear, and minimum micro-hardness. The samples with 5wt % RHA had minimum wear resistance and minimum density. 

The above literature confirms that no work has been reported on the investigation of the ternary reinforced AA7075 HMMC with SiC, RHA, and CES as primary, secondary, and tertiary reinforcements, respectively. Therefore, in this work, the mechanical and microstructural properties of a novel HMMC (AA7075-5wt %SiC-3wt %RHA-1wt %CES) were investigated. Four samples, including AA7075 (base alloy, sample 1), AA7075-5wt %SiC (MMC, sample 2), AA7075-5wt %SiC-3wt %RHA (s-HMMC, sample 3), and AA7075-5wt %SiC-3wt %RHA-1wt %CES (n-HMMC, sample 4) were developed using stir casting liquid metallurgy route, followed by the heat treatment. Finally, a comparative study of the heat-treated samples in terms of microstructural analysis, density and porosity, ultimate tensile strength, hardness, and impact strength were investigated.

## 2. Materials and Methods

**Figure 1:**
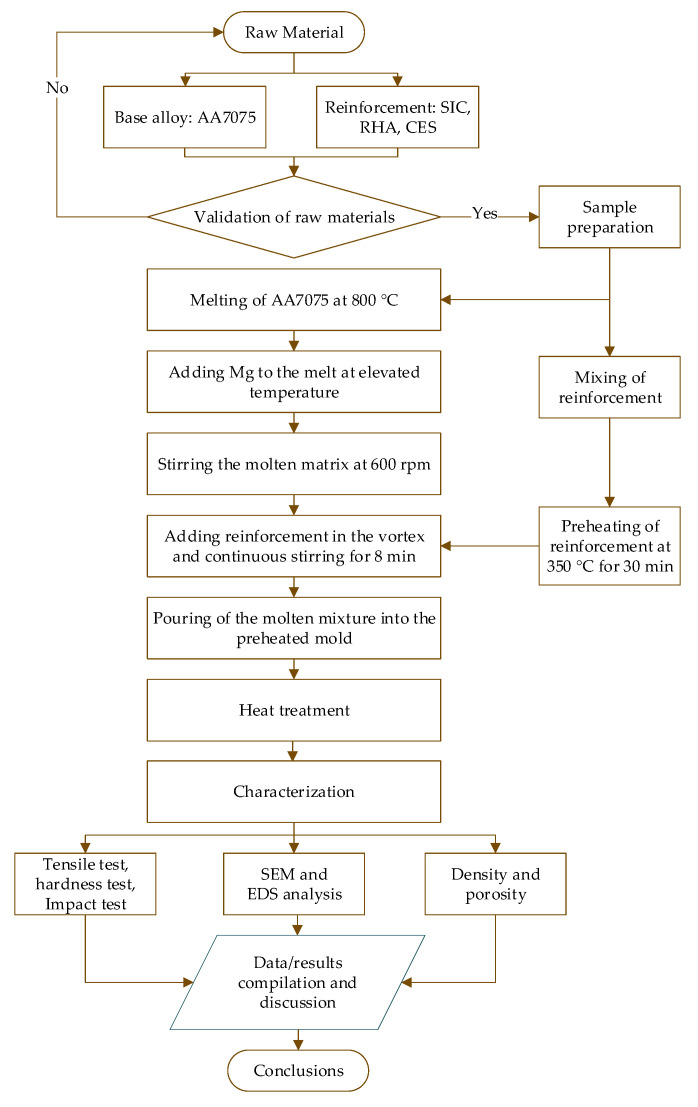
The flow chart of Methodology.

In this work, a novel ternary reinforced hybrid metal matrix composite was fabricated using a stir casting method. The base alloy AA7075 was used as a metal matrix, which was reinforced with 5wt % SiC, 3wt % RHA, and 1wt % CES particle. The casted composites were heat treated, followed by mechanical testing and microstructural analysis. The detailed methodology is presented in Figure 1.

### 2.1. Materials

**Figure 2:**
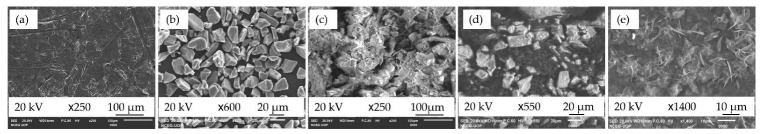
SEM of the raw materials (**a**) AA7075 (as received), (**b**) Silicon Carbide, (**c**) Rice Husk Ash, (**d**) Carbonized Eggshell and (**e**) Mg Ribbon.

AA7075 was used as a metal matrix, whereas SiC, RHA, and CES were used as reinforcing agents. The scanning Electron Microscopy (SEM) with Elemental Dispersive Spectroscopy (EDS) of each material was performed using the JEOL’s scanning electron microscope (Model: JSM-IT-100) to confirm the elemental composition of the materials. The SEM images of the raw materials are shown in Figure 2. The particle size reduction and uniform mixing of the reinforcements were carried out in the Gunt’s horizontal ball mill (Model: CE 245) at 200 rpm for 2 h. Beckman’s Multisizer 3 was used for the particle size analysis of the reinforcements.

**Figure 3:**
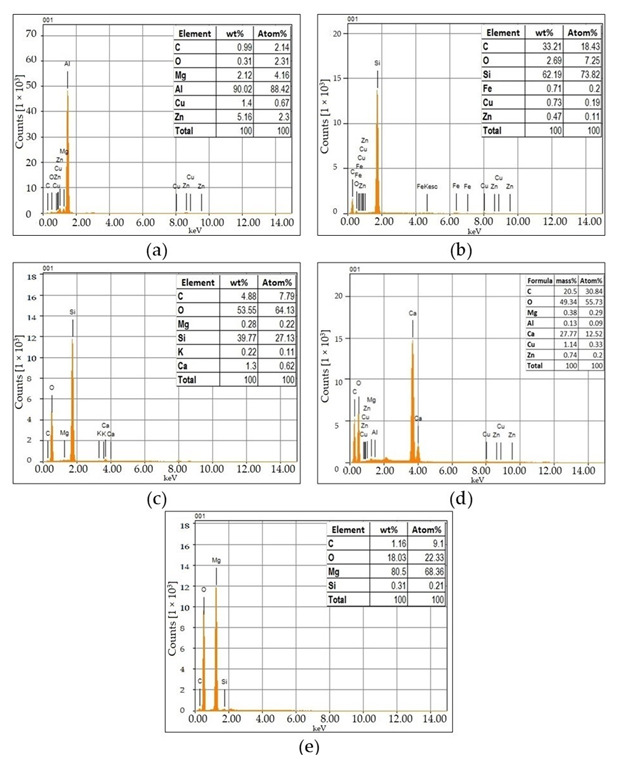
EDS of the raw materials (**a**) AA7075 (as received), (**b**) Silicon Carbide, (**c**) Rice Husk Ash, (**d**) Carbonized Eggshell ash (**e**) Mg Ribbon.

The aluminum alloy AA7075 was purchased in rod form from the local vendor, Mohammadi Metals, in Karachi—Pakistan. These rods were then cut into the required masses for further processing. The EDS results in Figure 3a validate that the material is AA7075 with 5.16wt % Zn as the major alloying element.

**Figure 4:**
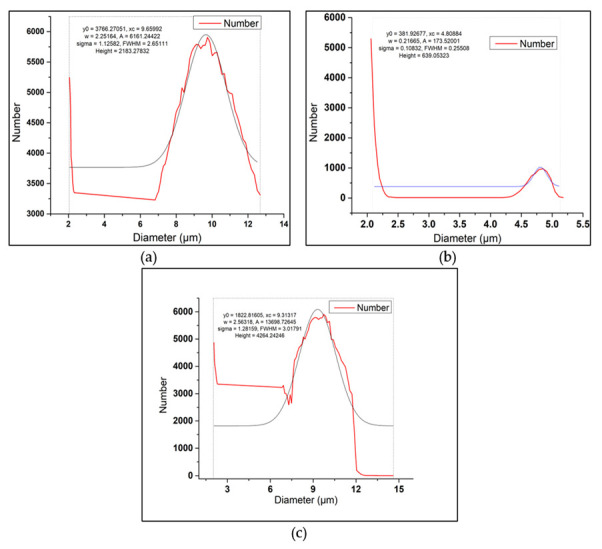
Particle size analysis curves of (**a**) Silicon Carbide powder (**b**) Rice Husk Ash, and (**c**) Carbonized Eggshell Powder. (Red line represents actual curve while the grey line represents Gaussian Curve Fit).

Silicon carbide was purchased from Haq Chemicals, Peshawar, Pakistan, in the form of powder with a mesh size of 800. EDS confirms the presence of Si and C in bulk (~90.6wt %), as given in Figure 3b. In addition, through the ball milling process, the average particle size of silicon carbide was reduced to 9.7 microns, as shown in Figure 4a, which was confirmed through the particle size analysis technique.

The risk husk was obtained from the local market in Pakistan, which was first cleaned for visible impurities and washed several times to clean off the dust, small impurities, and rotten husk residuals. The rinsed rice husk was dried for about 10 days at room temperature. The dried rice husk was heated in a graphite crucible in a Muffle furnace at 250 °C for 2 h to remove the moisture. Then it was heated to 600 °C at 10 °C/min and left at 600 °C for the next 24 h. During the conversion from rice husk to rice husk ash, its color changes from golden brown to black and ultimately to whitish powdered ash. The ash content was found to be 18 percent, which is in close agreement with the values mentioned in [51]. The presence of Si and O in bulk indicates the successful conversion of rice husk into the RHA, predicting more than 93.32wt % silica, Figure 3c. Similarly, the RHA was ground to a fine powder in the ball mill with an average particle size of 4.81 µm, Figure 4b, which was confirmed through the particle size analysis technique.

About 60 eggshells were collected from the local poultry farm in Peshawar–Pakistan. These eggshells were ground into a powder followed by carbonization at 500 °C for 4 h. To achieve a homogenous particle size, the carbonized eggshell powder was rotated in the ball mill at 200 rpm for 2 h to achieve an average particle size of about 9.31 microns, as evident from Figure 4c. In addition, the EDS of the CES is shown in Figure 3d, which confirms the presence of Ca, C, and O in bulk, indicating the existence of CaCO_3_.

Moreover, the magnesium ribbons were purchased from the local vendor, Haq Chemicals, Peshawar–Pakistan— and added to the melt as a filler material to increase the wettability of the molten mix of AA7075 matrix and the reinforcing materials. Figure 3e shows the EDS of the magnesium ribbon, which validates the presence of Mg in bulk. To achieve proper mixing, 1wt % of Mg ribbon was added to each sample except the base alloy. Additionally, argon gas with a 99.999% purity was bought from the local supplier–Khyber Oxygen (Pvt) Limited, Nowshera–Pakistan.

### 2.2. Sample Preparation

Aluminum alloy 7075 was melted in a graphite crucible of the stir casting machine at 800 °C with a constant heating rate of 5 °C/min. Next, Magnesium ribbons were added to the melt at elevated temperatures to increase the wettability between the molten matrix and reinforcements. Reinforcements were thoroughly mixed for 15–20 min in the ethanol environment using a horizontal ball mill. Pre-heating of the reinforcements was achieved at 350 °C for 30 min to remove the moisture content. The melt was then stirred at 600 rpm using two blades stainless steel stirrer. Upon the formation of the vortex, reinforcements were gradually added to the melt at the approximate rate of 1 gm/min. The supply of 99.9999% pure Argon gas was continuously maintained at 10 lit/min to avoid the formation of oxides during the mixing process. The stirring was performed continuously for about 8 min to achieve the proper and uniform mixing of the reinforcements into the melted matrix. Finally, the melt was poured into the pre-heated (480 °C) mold. It is worth noting that pre-heating of the mold was undertaken to avoid defects due to the sudden temperature difference between the melt and the mold. The samples were then allowed to gradually cool upon reaching room temperature and removed from the mold. 

The casted samples were AA7075 (Base alloy: Sample 1), AA7075-5wt %SiC composite (MMC: Sample 2), AA7075-5wt %SiC-3wt %RHA, secondary reinforced hybrid metal matrix composite (s-HMMC: Sample 3), and AA7075-5wt %SiC-3wt %RHA-1wt %CES, a novel hybrid metal matrix composite (n-HMMC: Sample 4). The compositional details in wt % of each sample are summarized in Table 1.

#### Heat Treatment

All of the casted samples were heat-treated by heating the samples at 400 °C for 3 h followed by quenching in a water bath at room temperature. Once quenched, the samples were heated to 450 °C for 2 h to achieve homogenization, followed by the aging process at 120 °C for 24 h.

### 2.3. Characterization and Mechanical Testing

#### 2.3.1. Scanning Electron Microscopy and Energy Dispersive Spectroscopy

All of the prepared samples were examined using JEOL’s Scanning electron microscope (Model: JSM-IT-100) for scanning electron microscopy (SEM) and Energy Dispersive Spectroscopy (EDS). SEM was performed to observe the surfaces and distribution of the reinforcements in the matrix. EDS was used for the elemental analysis of the samples to confirm the successful fabrication of the samples.

#### 2.3.2. Density and Porosity

The theoretical and experimental densities of the samples were investigated using Equation (1) of the rule of the mixture [52] and the Archimedes Principle (ASTM B962-13, [53]), respectively.

(1) $$\rho_{t}=\rho_{m}\;\emptyset_{m}+\rho_{r}\;\emptyset_{r}$$

where, $\rho_{t}$ $\rho_{m\;}$ and $\rho_{r}$ are the theoretical, matrix, and reinforcement densities, respectively. $\emptyset_{m}$ and $\emptyset_{r}$ are, respectively, the matrix and reinforcement weight fractions.

The percentage porosity of the samples was calculated using the following equation.

(2) $$\%porosity=\frac{\rho_{t}-\rho_{e}}{\rho_{t}}\times 100$$

where, $\rho_{t}$ and $\rho_{e}$ are the theoretical and experimental densities, respectively.

#### 2.3.3. Tensile Test

**Figure 5:**
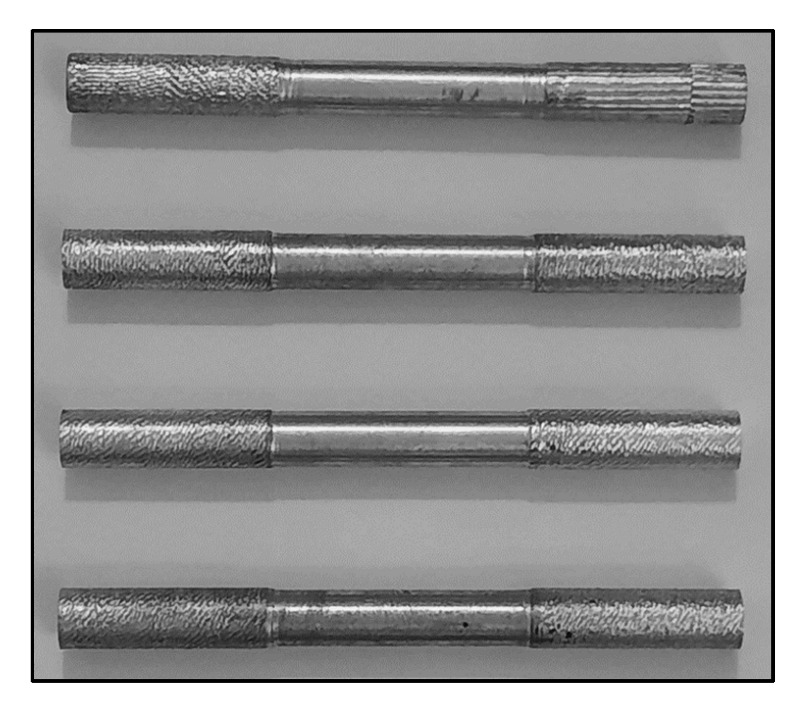
Casted samples machined into the tensile test specimen.

The tensile testing of each sample was performed on a Universal testing machine (Shimadzu’s AG-IS, Autograph, Japan, 100 KN) as per the ASTM B557-15 standard [54] with a specimen gauge length and gauge diameter of 2 in and $\frac{1}{2}$ in, respectively, Figure 5. The test was carried out at a crosshead speed of 2 mm/min, under room conditions (27 °C; Relative Humidity 60%).

#### 2.3.4. Hardness Test

The Rockwell (RH) and Brinell hardness (HBN) of the heat-treated samples were measured using the P.A. Hilton’s Rockwell/Brinell hardness tester (Model: HSM51 Rockwell/Brinell Combined System) as per ASTM E18-20 [55] and ASTM E10-18 standards [56]. Loads of 100 kgf and 187.5 kgf were applied for the total dwell time of 15 s, using steel ball indenters with diameters of 1.588 mm and 2.5 mm for the measurement of the Rockwell and Brinell hardness, respectively. Each sample was indented at four different points, and the average value was recorded.

#### 2.3.5. Impact Test

The impact test was performed using the procedure mentioned in the ASTM E23-16 standard [57]. Three V-notch samples of each composition were prepared for the impact test performed on the ESSOM’s Charpy Impact Tester Machine (Model: TM232, 25/50 J).

## 3. Results and Discussion

### 3.1. Density and Porosity

**Figure 6:**
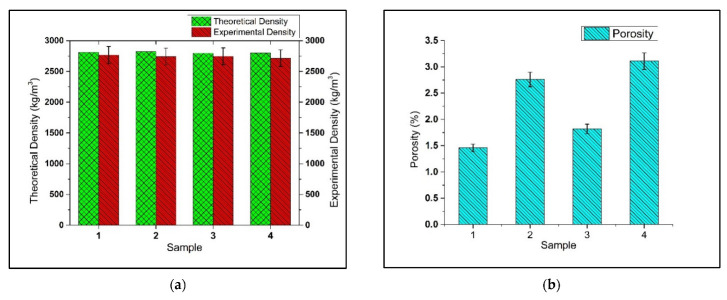
(**a**) Theoretical vs. experimental densities and (**b**) Porosity of: AA7075 (base alloy, sample 1), AA7075-5wt %SiC (MMC, sample 2), AA7075-5wt %SiC-3wt %RHA (s-HMMC, sample 3) and AA7075-5wt %SiC-3wt %RHA-1wt %CES (n-HMMC, sample 4).

The theoretical and experimental densities are compared in Figure 6a. The trend of experimental density follows the trend of theoretical density, which shows the successful fabrication of the samples. The results show that the maximum experimental density of AA7075 (Base alloy: Sample 1) was 2769 kg/m^3^. With an increasing number of reinforcements in the base alloy, a drop was recorded in the densities of the AA7075-5wt %SiC (MMC: Sample 2), AA7075-5wt %SiC-3wt %RHA (s-HMMC: Sample 3), and AA7075-5wt %SiC-3wt %RHA-1wt %CES (n-HMMC: Sample 4). The minimum experimental density of 2714 kg/m^3^ was recorded for n-HMMC, which is about 1.18% less than the base alloy. MMC and s-HHMC have also shown a marginal drop in their density compared to the base alloy. 

**Figure 7:**
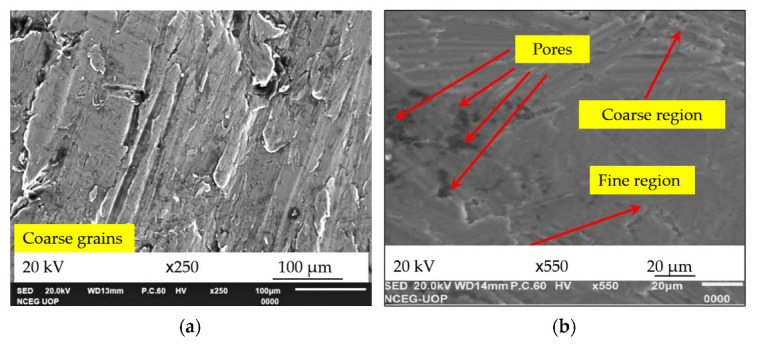
SEM image of (**a**) AA7075 (base alloy, sample 1), (**b**) AA7075-5wt %SiC-3wt %RHA-1wt %CES (n-HMMC, sample 4) agglomerations of reinforcements.

The percentage porosity of the reinforced composites was found to be greater than the base alloy (AA7075), Figure 6b. The results showed that n-HMMC has a maximum porosity of 3.11% compared to the 1.46% porosity of the base alloy. Similarly, MMC and s-HMMC have porosities of 2.76% and 1.82%, respectively. Hence, the n-HMMC showed the highest porosity following MMC, s-HMMC, and the base alloy. The same trend was mentioned in references [18,52,58,59]. The increase in porosity was due to the improper mixing of the reinforcement particles and the matrix, as shown in Figure 7b. Further, mixing reinforcements in the molten matrix at elevated temperatures increases the probability of oxide formation [18]. The factors that contribute to the increased porosity in MMCs and HMMCs include wettability issues [60], the temperature gradient between the matrix and reinforcement, non-uniform distribution of reinforcement particles in the matrix, the particle size of reinforcements, stirring speed, stirring time, and the pouring rate of the molten mixture into the mold [25,61,62,63,64,65].

### 3.2. Microstructural Examination

The samples for the microscopic examination were prepared as per the ASTM E3-11 standard [66]. Figure 7a shows that the base alloy AA7075 has coarse grains with minimum porosity. In contrast, Figure 7b confirms that n-HMMC has a mixture of fine grains, coarse grains, and some pores. It is worth noting that pores are most likely to be due to improper mixing, pouring issues, and trapped gases. CaCO_3_ was the major constituent of CES, which releases CO_2_ gas when it reacts with oxygen. As a result, CO_2_ escapes the molten mixture, leaving behind the pores, thus producing a foaming effect.

**Figure 8:**
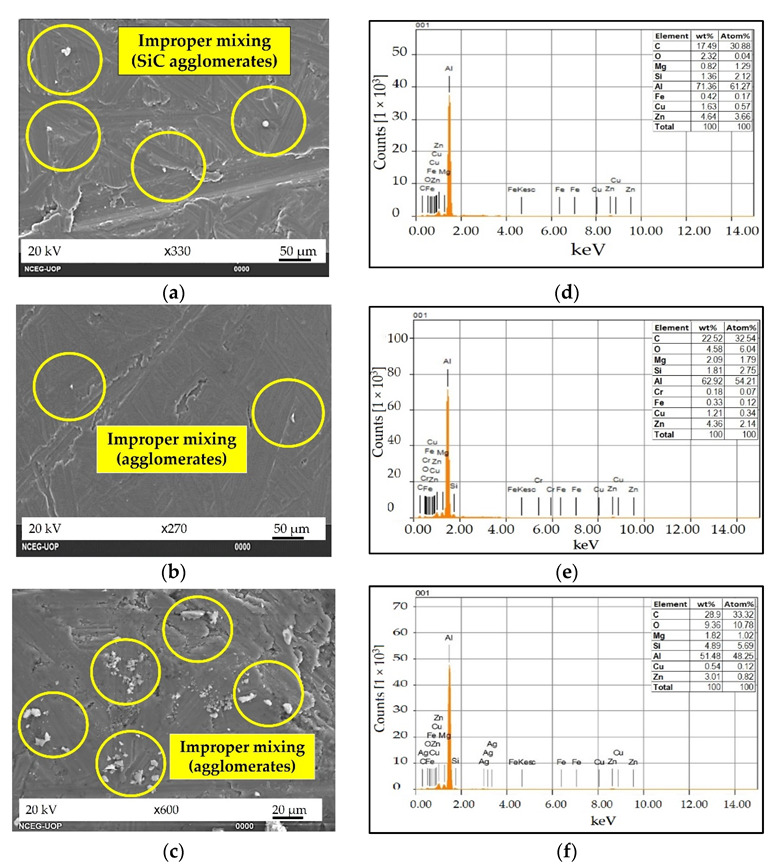
SEM image of: (**a**) AA7075-5wt %SiC (MMC, sample 2), (**b**) AA7075-5wt %SiC-3wt %RHA (s-HMMC, sample 3) (**c**) AA7075-5wt %SiC-3wt %RHA-1wt %CES (n-HMMC, sample 4) and EDS of: (**d**) AA7075-5wt %SiC (MMC, sample 2), (**e**) AA7075-5wt %SiC-3wt %RHA (s-HMMC, sample 3), (**f**) AA7075-5wt %SiC-3wt %RHA-1wt %CES (n-HMMC, sample 4).

MMC and s-HMMC have shown the uniform distribution of the particles in the matrix, as indicated in Figure 8a,b. The former has shown some agglomerates of SiC in the matrix material, Figure 8a. However, it has been observed that the addition of the 3wt % RHA into the MMC to form s-HMMC improves the distribution of the particles in the matrix. It is most likely due to the reduced particle size of the RHA and better hybrid mixture formation capabilities of SiC and RHA in the molten mixture. Hence, due to its fine size, RHA showed a greater capability to form a uniform distribution in the matrix, which leads to negligible agglomeration. 

On the contrary, n-HMMC has shown mixing issues, and the agglomerations of reinforcements in the matrix have been observed in Figure 8c. These agglomeration sites are treated as stress concentration points that can weaken the material, especially under impact loading [7,18]. Further, it has been observed that the addition of 1% CES as tertiary reinforcement has caused an increase in porosity and agglomerations due to the improper mixing of the ingredients in the matrix. The wettability issues increase as the number of constituting reinforcement particles increases. However, despite the increase in the porosity and agglomerations, n-HMMC has achieved more refined grains than the base alloy, Figure 7a, and MMC, Figure 8a. This is the possible reason for its highest hardness and UTS. As a generic trend, it has been observed that the increasing number of reinforcements in the matrix results in more refined grains but leads to embrittlement and agglomerations. Agglomerations can be avoided with more improved fabrication processes and controlled parameters. The EDS of MMC, s-HMMC, and n-HMMC are shown in Figure 8d–f, while the EDS of the base alloy was already shown in Figure 3a.

### 3.3. Mechanical Properties

#### 3.3.1. Tensile Properties 

**Figure 9:**
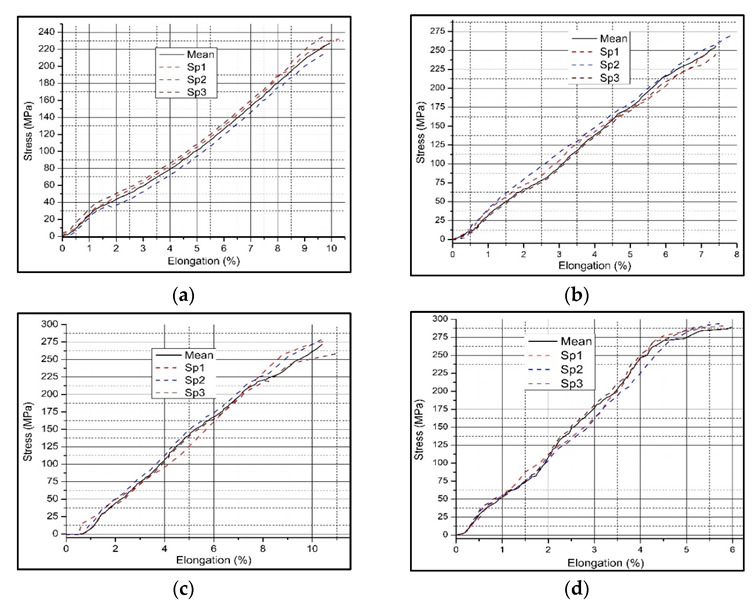
Stress–Strain relation of: (**a**) AA7075 (base alloy, sample 1), (**b**) AA7075-5wt %SiC (MMC, sample 2), (**c**) AA7075-5wt %SiC-3wt %RHA (s-HMMC, sample 3) and (**d**) AA7075-5wt %SiC-3wt %RHA-1wt %CES (n-HMMC, sample 4).

**Figure 10:**
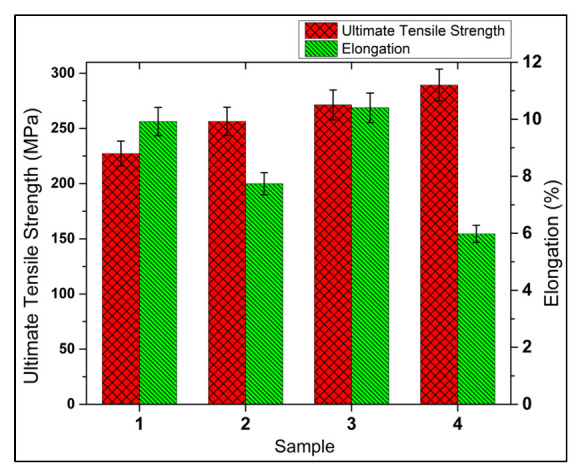
Average valves of ultimate tensile strength and percentage elongation of: AA7075 (base alloy, sample 1), AA7075-5wt %SiC (MMC, sample 2), AA7075-5wt %SiC-3wt %RHA (s-HMMC, sample 3) and AA7075-5wt %SiC-3wt %RHA-1wt %CES (n-HMMC, sample 4).

In this study, the tensile properties were analyzed in terms of the ultimate tensile strength (UTS) and the percentage elongation (%EL), as shown in Figure 9. The results showed that the addition of ceramic reinforcing particles increased the strength of the base alloy. The n-HMMC showed the maximum UTS value of 289.26 MPa, which is about 24.4%, 11.4%, and 6.2% higher than the base alloy, MMC, and s-HMMC, respectively. In contrast to the UTS, n-HMMC showed the highest reduction in ductility with the least value of 5.98%. Figure 10 summarizes the average valves of the UTS and %EL. 

According to Hall–Petch theory, this increase in the UTS of n-HMMC is most likely because of the more refined grains achieved due to the increased number of nucleation sites available and the partial uniform mixing of the reinforcements in the matrix [67,68]. However, the increase in embrittlement is related to the increase in the ceramic content, improper mixing, and agglomeration of the reinforcing particles, as confirmed by Figure 8c. The maximum ductility of 10.4% is recorded for the s-HMMC with a higher strength of 271.36 MPa. An increase in the ductility and strength of the s-HMMC is due to the fine size of the RHA, which results in the formation of more refined grains in the s-HMMC Figure 8b, which is the main reason for its maximum ductility. An almost similar behavior of the addition of the RHA to the AA7075 matrix has been reported by Verma and Vettivel [41]. This confirms that the proper mixing and uniform distribution of the reinforcing particles in s-HMMC have overcome the adverse effect of the increasing ceramic content and led to optimum tensile properties. n-HMMC, despite its highest UTS, has shown compromised ductility, which is most likely due to the dominating effect of the high ceramic content.

#### 3.3.2. Hardness

**Figure 11:**
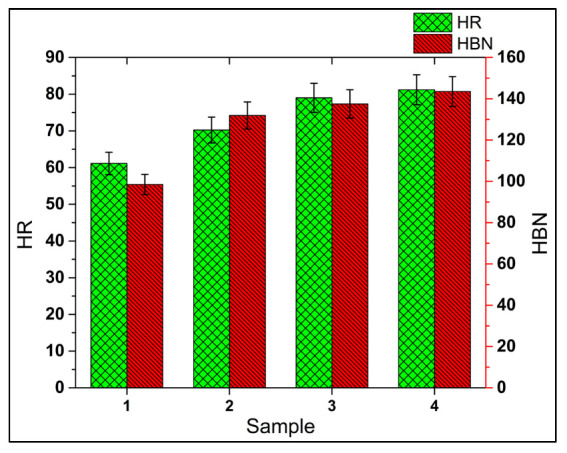
Hardness of: AA7075 (base alloy, sample 1), AA7075-5wt %SiC (MMC, sample 2), AA7075-5wt %SiC-3wt %RHA (s-HMMC, sample 3) and AA7075-5wt %SiC-3wt %RHA-1wt %CES (n-HMMC, sample 4).

The results of the Rockwell (HR) and Brinell (HBN) hardness are presented in Figure 11. An increasing trend is observed in the hardness values as the number of reinforcements increases. Hence, n-HMMC has the maximum hardness of HR = 81.2; HBN = 143.55, whereas the base alloy AA7075 showed the minimum value, HR = 61.13; HBN = 98.5. n-HMMC, s-HMMC, and MMC have shown a 32.83%, 29.23%, and 14.89% increase in the Rockwell hardness compared to its base alloy. These results are in good agreement with references [9,18,41]. The increasing trend in the hardness of the composite materials with the increasing ceramic reinforcements was due to the Orowan mechanism in which there is an increase in the dislocation–reinforcement interaction hence providing an overall increase in the hardness [69]. Additionally, the heat treatment and subsequent aging of the samples release the residual stresses, which also improve the hardness of the material [70,71]. Despite the agglomerations and partially uniform distribution of the reinforcement particles, the increase in the hardness value of n-HMMC has confirmed the dominating effect of the ceramic portion over the adverse effects of the agglomeration, as evident from Figure 8c. Hence, the SEM surface analysis of the fabricated samples reveals that n-HMMC has the maximum ceramic reinforcement portion compared to the base alloy, MMC, and s-HMMC.

#### 3.3.3. Impact Strength

**Figure 12:**
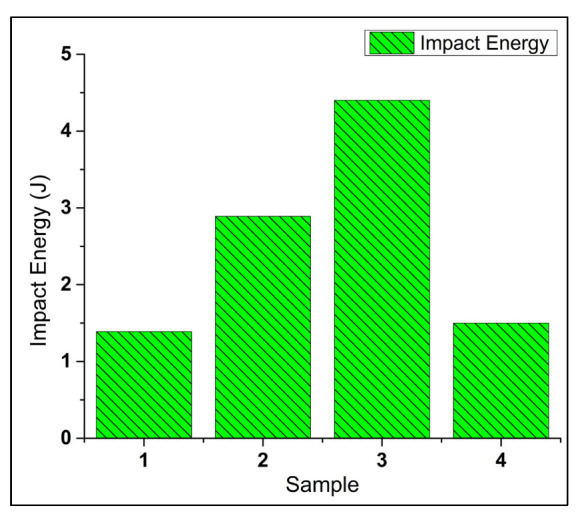
Average Impact Energy of: AA7075 (base alloy, sample 1), AA7075-5wt %SiC (MMC, sample 2), AA7075-5wt %SiC-3wt %RHA (s-HMMC, sample 3), and AA7075-5wt %SiC-3wt %RHA-1wt %CES (n-HMMC, sample 4).

Figure 12 shows the average impact strength of all the samples. The results showed that with the addition of 5wt % SiC to the matrix in MMC and the addition of 3wt % RHA in s-HMMC, the impact strength increased by 106.4% and 214.3% compared to the base alloy, respectively. s-HMMC has the maximum average impact energy of 4.4J due to the reduced particle size of RHA, which has achieved a more uniform distribution of reinforcements, and an increased number of nucleation sites, providing a strengthening effect. It was also observed that SiC and RHA showed a minimum tendency to form agglomerates when mixing in the molten matrix of the base alloy (AA7075) in the ratio of 5:3 by weight. Upon the addition of the 1wt % CES particles along with 5wt % SiC and 3wt % RHA, n-HMMC showed a drop of about 66% and 47.7% in the impact strength as compared to MMC and s-HMMC, respectively. However, it has a marginal increase of around 8.6% compared to the base alloy. The drop in the impact strength of the n-HMMC is due to the increased porosity, Figure 7b, improper mixing of the reinforcements in the matrix, and the formation of agglomerates, as evident from Figure 8c. This is because the agglomerations create more vulnerable sites for failure under the impact loading.

## 4. Conclusions

This work is focused on the investigation of the mechanical and microstructural properties of the novel hybrid metal matrix composite (n-HMMC) AA7075-5wt %SiC-3wt %RHA-1wt %CES, prepared via a stir casting process. A comparative study of n-HMMC was conducted with its base alloy (AA7075), primary reinforced metal matrix composite (MMC) AA7075-5wt %SiC, and the secondary reinforced hybrid metal matrix composite (s-HMMC) AA7075-5wt %SiC-3wt %RHA. Based on the investigation, the following conclusions can be drawn:

- The highest ultimate tensile strength of 289 MPa was obtained for n-HMMC (AA7075-5wt %SiC-3wt %RHA-1wt %CES), which was 24.4%, 12.8%, and 6.6% higher than the base alloy, MMC and s-HMMC, respectively;
- The highest hardness of RH = 81.2 was recorded for n-HMMC, which was 32.83%, 29.23%, and 14.9% higher than the base alloy, MMC, and s-HMMC, respectively;
- The density of the n-HMMC was 1.18% less than the density of the base alloy, which has an experimental density of 2769 kg/m^3^. Consequently, among all the samples, n-HMMC has the highest porosity of 3.25% due to improper mixing of reinforcements and matrix, wettability issues, agglomerations, and pouring defects;
- The highest %EL of 10.4% was observed in s-HMMC (AA7075-5wt %SiC-3wt %RHA). On the other hand, n-HMMC has the least ductility at 5.98%. The reduction in ductility is due to the dominating effect of the ceramic particles’ dispersion over the reinforcement-induced grain refinement effect;
- MMC and s-HMMC showed a greater increase in the impact energy of about 106% and 214%, respectively, as compared to the base alloy. However, a marginal increase of 8.6% was observed in the impact energy of the n-HMMC. The drop in impact strength with the addition of tertiary reinforcement (1wt % CES) was due to the non-uniform distribution of reinforcements, wettability issues, and the formation of the agglomerates;
- EDS analysis confirmed the presence of the respective elements of the reinforcements in bulk for the successful preparation of samples through the stir casting process. SEM analysis shows the relatively uniform distribution of the reinforcing particles in the MMC and s-HMMC. However, higher agglomerates are observed in the n-HMMC due to the increase in the ceramic content and mixing issues. These agglomerates serve as the potential site of stress concentration, compromising the ductility and impact strength;
- Though stir casting is one of the commonly used processes for fabricating MMCs and HMMCs; however, more controlled process parameters are required to avoid mixing issues, agglomerates, and oxides formation. Therefore, it is recommended to develop the same n-HMMC with different fabrication techniques to address the aforementioned shortcomings of the stir casting technique, if possible;
- In the future, the findings of this research could be used for practical applications in aerospace, automobile, defense, and marine industry for which its processibility, machining, and in-service capabilities are required for further investigations.

## Figures and Tables

**Table 1:** The composition of casted samples.

Sample	Composition (wt %)				
	AA7075	Mg	SiC	RHA	CES
	Base Alloy	Filler Material	Primary Reinforcement	Secondary Reinforcement	Tertiary Reinforcement
1	100	0	0	0	0
2	94	1	5	0	0
3	91	1	5	3	0
4	90	1	5	3	1

## Data Availability

Not applicable.
